# CULTURAL ADAPTION OF THE INTERNET-BASED CONVERSATIONAL CLINICAL TRIAL (I-CONECT) FOR ASIAN OLDER ADULTS

**DOI:** 10.1093/geroni/igae098.2267

**Published:** 2024-12-31

**Authors:** Kexin Yu, Amanda Mar, Minju Song, Qijia Hu, Shiyun Chen, Christine Le, Mia Huynh, Hiroko Dodge

**Affiliations:** Oregon Health and Science University, Portland, Oregon, United States; Oregon Health & Science University, Portland, Oregon, United States; Portland State University, Portland, Oregon, United States; University of Illinois Urbana-Champaign, Champaign, Illinois, United States; New York University, New York, New York, United States; Oregon State University, Corvallis, Oregon, United States; Reed College, Portland, Oregon, United States; Harvard Medical School, Massachusetts General Hospidal, Framingham, Massachusetts, United States

## Abstract

Alzheimer’s disease and related dementias (ADRD) are influenced by cultural and social factors. Asian older adults with limited English proficiency often face the risk of social isolation, which is recognized as a risk factor for the incidence of ADRD. However, few behavioral health interventions for dementia prevention have been designed specifically for the Asian American population. The Internet-Based Conversational Clinical Trial (I-CONECT, ClinicalTrials.gov Identifier: NCT02871921) examined whether conversations through user-friendly video chats could enhance cognitive functions and emotional well-being. Although the previous study showed efficacy, it was limited to predominantly white Caucasian participants. This presentation will describe the cultural and linguistic adaptation process of I-CONECT for older Asian adults. A mixed-methods approach was used, including 1) conducting semi-structured interviews with older Asian adults to qualitatively understand the lived experience of social isolation to inform culturally competent intervention inclusion criteria and 2) translating and adapting the intervention material to be culturally competent and pilot testing it with 10 participants. Participants will have four times/week conversation sessions with trained research staff for a 6-week intervention period. The pre- and post-intervention assessments include loneliness, social isolation, and cognitive test scores. Participants will also elaborate on their experience in post-intervention qualitative interviews. The feasibility and acceptance of online conversation interventions have not been empirically tested with Asian Americans. The cultural adaptation of an existing evidence-based intervention model could lay the foundation for expanding effective behavioral health interventions for preventing ADRD in the underrepresented population.

